# Revisiting price linkages between London and Shanghai base metal futures markets: A time-frequency connectedness analysis

**DOI:** 10.1371/journal.pone.0346602

**Published:** 2026-04-09

**Authors:** Cunhai Pan, Wei Shen

**Affiliations:** School of Earth Sciences and Resources, China University of Geosciences (Beijing), Beijing, China; Bilecik University: Bilecik Seyh Edebali Universitesi, TÜRKIYE

## Abstract

Against the backdrop of recent global events (including the COVID-19 pandemic, the Russia-Ukraine war, and escalating trade tensions), this paper revisits price linkages between the London Metal Exchange (LME) and the Shanghai Futures Exchange (SHFE) base metal futures markets. Utilizing a Time-Varying Parameter Vector Autoregressive (TVP-VAR) frequency-based connectedness approach, we analyze daily data (2017–2024) to uncover dynamic spillovers across copper, nickel, aluminum, and zinc. The results demonstrate that the LME maintains its role as the global pricing benchmark, particularly for copper (42.82% contribution to SHFE variance), while the SHFE remains a net receiver, with short-term effects dominating (e.g., the SHFE market absorbs 36.87% of the short-term net spillover effects). Despite China’s internationalization efforts, the SHFE’s price leadership has declined relative to its historical position, reflecting the combined effects of domestic structural adjustments, institutional frictions, and major global shocks. Both markets exhibit event-specific sensitivities: the LME’s influence surged during supply chain crises (e.g., 2022 nickel short squeeze), while the SHFE’s responsiveness is constrained by institutional barriers. These findings underscore the LME’s role as a global pricing benchmark and urge policymakers in emerging economies to enhance market resilience through regulatory coordination, market development, and improved market transparency.

## 1. Introduction

Raw materials serve as the backbone of the global economy. Over the past few decades, China has emerged as the largest consumer and producer of numerous base metals (also referred to as non-ferrous or industrial metals) globally [1–3]. On the supply side, global mineral production reached 1.87 billion tons in 2022 [4]. Among the leading mining countries, China, the USA, Russia, and Australia ranked as the top four, accounting for 26.4%, 12.2%, 8.5%, and 6.9% of total production, respectively [4]. Notably, China is the world’s foremost producer of 28 key commodities, highlighting its substantial impact on the global base metals supply. In terms of demand, China’s metal consumption accounts for 60% of the global total, driven by sectors such as infrastructure and manufacturing, including electric vehicles [5]. This dual dominance positions China as a key player in shaping both physical and financial metal markets. Kang and Yoon [2] argued that China’s economic activities play a significant role in determining global non-ferrous metal prices. Moreover, China’s financial influence is reflected in the Shanghai Futures Exchange (SHFE), which has evolved into the world’s second-largest non-ferrous metal futures platform, trailing only the London Metal Exchange (LME), driven by progressive regulatory reforms [6,7].

Given China’s pivotal role, the price linkage between the SHFE and the global benchmark LME has been a central focus of academic inquiry. Early research consistently identified the LME as the dominant price discovery center, with studies like Fung et al. [8] and Li and Zhang [9] documenting unidirectional spillovers from international markets to China for key commodities like copper. This early asymmetry was often attributed to the immaturity and lower efficiency of China’s nascent futures market [10]. As the SHFE matured, evidence of a more complex, bidirectional relationship emerged. For instance, Liu and An [11] found bidirectional spillovers between the U.S. and Chinese markets, while direct LME-SHFE studies confirmed long-run cointegration [12]. Notably, some research began to capture moments of shifting influence, such as the SHFE temporarily becoming a net volatility transmitter during the European debt crisis [2], and the identification of bidirectional risk spillovers using more sophisticated dynamic models [1,13,14]. Furthermore, the literature recognizes significant heterogeneity across different metals, with studies highlighting varying co-dependence patterns for aluminum [15], and copper [16].

While this body of work provides a valuable foundation, it leaves critical gaps that our study aims to address—particularly in the context of the turbulent period post-2017. First, there is a methodological gap. Prior studies relying on static correlations or traditional VAR/GARCH frameworks are ill-equipped to disentangle the transient, high-frequency spillovers (often driven by noise and speculative trading) from the persistent, low-frequency spillovers (reflecting long-term structural linkages). The time-frequency connectedness approach pioneered by Baruník and Křehlík [17] is precisely designed for this purpose but has not been fully applied to dissect the dynamic leadership between the LME and SHFE. Second, and more importantly, there is a substantive empirical and explanatory gap. The existing literature offers limited insights into the cumulative impact of the successive, overlapping global shocks since 2017—namely the Sino-US trade conflict, the COVID-19 pandemic, and the Russia-Ukraine war. These events have fundamentally altered the global geopolitical and economic landscape established after World War II, potentially reshaping the very foundations of market linkages [18]. The rise in trade protectionism, coupled with increased concerns over mineral supply chain security, has prompted economies such as the United States, Europe, and China to successively compile inventories of critical minerals. These uncertainties signal the potential end of an era characterized by the most efficient global free allocation of resources, with resource security emerging as a key factor in the formulation of resource policies by different economies. For instance, Ding et al. [19] argued that the Sino-US trade conflict caused significant fluctuations and even mutations in causal relationships within metal markets. Similarly, Chen and Tongurai [20] found significant structural breaks in the dynamic correlations among global commodity exchanges during the pandemic. In this new era of heightened uncertainty and resource nationalism, the historical patterns of price leadership are likely to have been disrupted.

Against this backdrop, whether the SHFE’s price leadership has weakened in the post-2017 period emerges as a critical empirical question. If such a decline exists, is it a temporary phenomenon or a new normal, driven by a combination of domestic challenges (e.g., China’s economic slowdown and real estate adjustments) and a less hospitable global environment? The existing literature, which primarily focuses on single events or static periods, cannot provide a satisfactory answer.

To bridge these gaps, this study revisits price spillovers between the London and Shanghai base metal futures markets for the four most actively traded base metals—copper, nickel, aluminum, and zinc—using a Time-Varying Parameter Vector Autoregressive (TVP-VAR) frequency connectedness framework. We focus exclusively on the bilateral linkage between the LME and SHFE, driven by two main considerations: First, they represent the world’s largest and fastest-growing base metal futures exchanges, making their interaction of paramount importance to the global industry; Second, this focused approach allows for a deeper, more granular analysis of the dynamic and frequency-specific spillovers between these two core price discovery hubs, which might be diluted in a more complex multi-market system. Moreover, extensive prior studies have specifically examined the connectedness between these two markets (e.g., [1,2,12,15]), further underscoring their significance. Our analysis spans January 2017 to June 2024, explicitly encompassing the three major events mentioned above.

This study contributes to the literature in three ways: First, we methodologically advance the literature by providing a dynamic, time-frequency decomposition of price leadership, effectively separating short-term noise from long-term structural spillovers. Second, we evaluate the heterogeneous impacts of three major global events (2017–2024), providing empirical evidence on how geopolitical and economic shocks reshape market linkages across different frequencies. Concurrently, we tested the hypothesis that the price leadership landscape has undergone a structural shift since 2017. Third, we identify the interplay of global geopolitical shocks and domestic structural changes as a novel mechanism explaining the SHFE’s declining influence—a dimension absent in existing studies focused solely on macroeconomic factors.

The remainder of the study is structured as follows: Section 2 outlines the methodology; Section 3 presents the data and preliminary findings; Section 4 discusses the empirical results; and Section 5 concludes.

## 2. Methodology

The systemic connectedness (spillover) between markets can be effectively analyzed through variance decompositions derived from a vector autoregression (VAR) model, which are intrinsically linked to modern network theory and systemic risk measures [21–23]. To overcome the limitations of static models and to disentangle short-term noise from long-term structural linkages, this study employs the Time-Varying Parameter VAR (TVP-VAR) based frequency connectedness framework introduced by Baruník and Křehlík [17] and advanced by Antonakakis et al. [24]. This approach is superior to traditional fixed-parameter or rolling-window VAR models as it does not require the arbitrary selection of a window size, thereby avoiding the associated loss of information and subjectivity. More importantly, it allows for the dynamic decomposition of connectedness across different frequency bands, which is crucial for understanding how shocks at various investment horizons transmit between the LME and SHFE.

### 2.1. TVP-VAR frequency connectedness framework

We begin with a TVP-VAR(*p*) model of the form:


$$y_{t}=\emptyset_{t,\text{1}}y_{t-\text{1}}+\emptyset_{t,\text{2}}y_{t-\text{2}}+\ldots +\emptyset_{t,p}y_{t-p}+\varepsilon_{t},\;\varepsilon_{t}\sim N(\text{0},\sum_{t})$$ (1)

where *y*_*t*_ is an *N × 1* vector of endogenous variables. In this study, *𝑁 =* 8, and the variables are ordered as *y*_*t*_ *=* (*Cu_LME, Ni_LME, Al_LME, Zn_LME, Cu_SHF, Ni_SHF, Al_SHF, Zn_SHF*),’ where each element denotes the daily logarithmic return of the corresponding metal futures series. The matrices *Φ*_*t,i*_ and *Σ*_*t*_ denote the time-varying coefficient matrix and variance-covariance matrix of the disturbances *ε*_*t*_, respectively. The model is estimated within a Bayesian TVP-VAR framework, which is well suited to capturing parameter instability and time variation in cross-market spillovers [25]. In the empirical implementation, the estimation is conducted in R using the *ConnectednessApproach* package [26]. The lag length *p* is set to 1 based on the Schwarz Information Criterion (SIC). To analyze connectedness in the frequency domain, the TVP-VAR model is first transformed to its time-varying vector moving average (TVP-VMA) representation:


$$y_{t}=\Psi_{t}(L)\varepsilon_{t}$$ (2)

where *Ψ*_*t*_(*L*) = *ψ*_*ij,t*_(*L*) is the time-varying lag polynomial. This representation is the cornerstone for spectral analysis.

### 2.2. Frequency generalized forecast error variance decomposition

The core of our analysis rests on the Frequency Generalized Forecast Error Variance Decomposition (GFEVD), which combines the spectral density of *y*_*t*_ with the generalized variance decomposition. It quantifies the proportion of the forecast error variance of variable *i* at a specific frequency *ω* that can be attributed to shocks in variable *j*. The non-normalized GFEVD is given by:


$$\begin{array}{c} \theta_{ijt}(\omega )=\frac{\overset{-\text{1}}{\underset{jj}{\left(\Sigma_{t}\right)}}{|\sum_{h=\text{0}}^{\infty }\;{\left(\Psi_{t,h}e^{-i\omega h}\right)}_{ij}|}^{\text{2}}}{\sum_{h=\text{0}}^{\infty }\;{\left(\Psi_{t,h}e^{-i\omega h}\Sigma_{t}{\Psi^{\prime }}_{t,h}e^{i\omega h}\right)}_{ii}} \end{array}$$ (3)

where *Ψ*_*t,h*_ is the *h-th* coefficient matrix of the TVP-VMA representation at time *t*. The term *e*^*−iωh*^ is the Fourier transform component that translates the time-domain impulse responses *Ψ*_*t,h*_ to the frequency domain at frequency *ω*. The denominator represents the total power (variance) of the spectrum of variable *i* at frequency *ω*.To enable a meaningful interpretation across variables, the GFEVD is normalized such that the contributions sum to one for each variable *i*:


$${\overset{\sim }{\theta }}_{ij,t}(\omega )=\;\frac{\theta_{ij,t}(\omega )}{\sum_{k=\text{1}}^{N}\theta_{ik,t}(\omega )}$$ (4)

Here, ${\overset{\sim }{\theta }}_{ij,t}(\omega )$ represents the intra-frequency connectedness. It measures the fraction of the spectrum of the *i-th* variable at frequency *ω* that can be ascribed to a shock in the *j-th* variable, relative to all shocks affecting *i* at that same frequency.

### 2.3. Aggregation over frequency bands and connectedness measures

To evaluate connectedness over specific time horizons relevant to market participants, we aggregate the spectral contributions over designated frequency bands. Let *d* = (*a, b*): *a, b ∈* (*−π, π*), *a* < *b* define a frequency band. The aggregated spillover from *j* to *i* within band *d* is:


$${\overset{\sim }{\theta }}_{ij,t}(d)=\int_{a}^{b}{\overset{\sim }{\theta }}_{ij,t}(\omega )\;d\omega$$ (5)

In accordance with established conventions in the literature and recognizing the distinct investment horizons of market participants—specifically, short-term arbitrageurs/speculators and long-term institutional investors/hedgers—we delineate two bands. The short-term band, spanning 1–5 days, corresponds to the frequency interval (*π, π/5*), whereas the long-term band, comprising periods exceeding 5 days, corresponds to the frequency interval (*π/5, 0*). This bifurcation allows us to disentangle noise from structural shifts, a critical advantage over static models. Various types of frequency connectedness measures, which provide information on spillovers within a specific frequency range *d*, can be computed as follows:

Total directional spillover received “FROM” others by market *i*:


$$FROM_{it}(d)=\sum_{j=\text{1},i\neq j}^{N}\;{\tilde{\theta }}_{ijt}(d)$$ (6)

Total Directional Spillover Transmitted “TO” others by market *i*:


$$TO_{it}(d)=\sum_{j=\text{1},i\neq j}^{N}\;{\tilde{\theta }}_{jit}(d)$$ (7)

*NET* total directional spillover of market *i*:


$$NET_{it}(d)=TO_{it}(d)-FROM_{it}(d)$$ (8)

A positive (negative) *NET* indicates that market *i* is a net transmitter (receiver) of spillovers within band *d*. Net pairwise directional connectedness (*NPDC*) from market *j* to market *i*:


$$NPDC_{ij,t}(d)={\tilde{\theta }}_{ij,t}(d)-{\tilde{\theta }}_{ji,t}(d)$$ (9)

This measure refines the analysis to the bilateral relationship level. Total connectedness index (*TCI*) of the system:


$${TCI}_{t}(d)=\frac{\sum_{i=\text{1}}^{N}{TO}_{it}(d)}{N}=\frac{\sum_{i=\text{1}}^{N}{FROM}_{it}(d)}{N}$$ (10)

The *TCI* measures the system-wide interconnectedness, or the average spillover from one market to all others, within the frequency band *d*. The overall *TCI* (across all frequencies) is given by *TCI*_*t*_ (*π*).

Software Implementation: The empirical analysis was conducted in R version 4.3.3 using the *ConnectednessApproach* package [26]. The connectedness measures were estimated using the TVP-VAR model with frequency connectedness specification. Following the package implementation, the lag order was set to *nlag* = 1, and the forecast horizon for the generalized forecast error variance decomposition was set to *nfore* = 100. The prior specification was set to *BayesPrior*, with forgetting factors kappa1 = 0.99 and kappa2 = 0.99. Generalized variance decomposition was adopted (*generalized* = *TRUE*) to ensure that the results were invariant to variable ordering. For the frequency decomposition, the partition was specified as (*π* + 0.00001, *π*/5, 0), corresponding to the short-term (1–5 days) and long-term (more than 5 days) connectedness components reported in this study.

## 3. Data and preliminary analysis

### 3.1. Data

This paper examines the return spillovers of the four most actively traded base metals—copper (Cu), nickel (Ni), aluminum (Al), and zinc (Zn)—on the LME and the SHFE from January 3, 2017, to June 28, 2024. We obtain 1,770 observations by accessing daily data from the two best-matched datasets: LME 3-month futures (electronic) and SHFE dominant-continuous futures contracts (which are traded continuously and in the largest volumes), with a focus on closing prices. Non-trading days and holidays are excluded to ensure synchronous data alignment between the LME and SHFE. All price time series are retrieved from the WIND Commodity Database, and log returns are calculated by taking the first differences of the log-transformed data.

### 3.2. Preliminary

**Fig 1:**
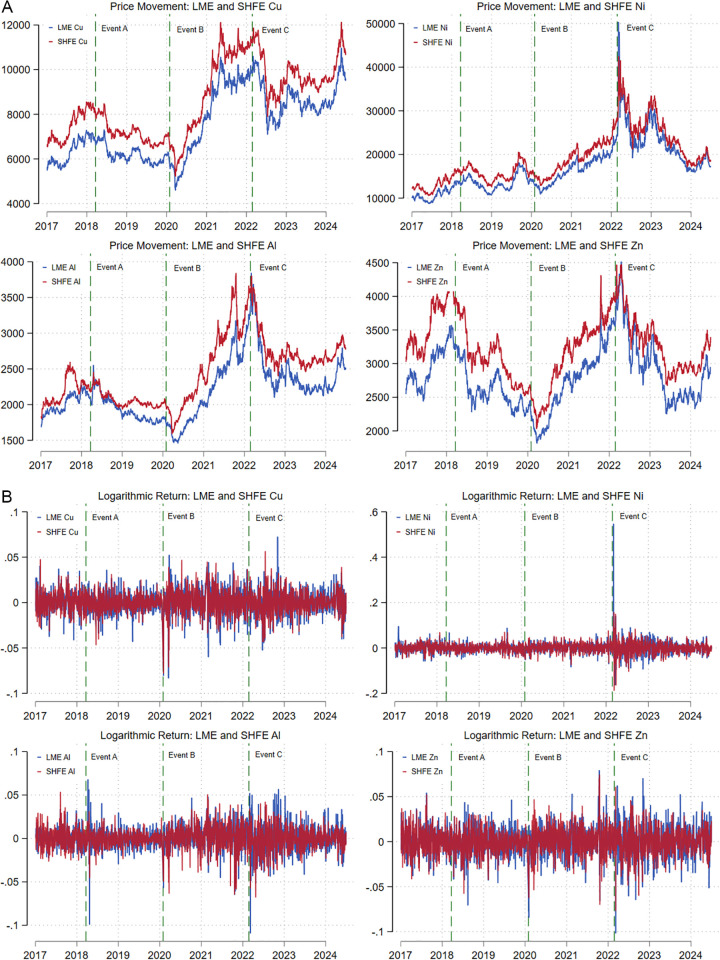
Daily closing prices and logarithmic returns for base metals on the LME and SHFE. A. Daily closing prices (in USD/ton) for base metals on the LME and SHFE. Notes: The dark blue and dark red lines represent the evolution of 3-month futures closing prices and dominant-continuous futures contract prices for four base metals (Cu, Ni, Al, and Zn) on the LME and SHFE, respectively. The vertical dashed lines mark the onset of major events: A (Sino-US trade conflict), B (COVID-19 pandemic), and C (Russia-Ukraine war). B. Corresponding logarithmic returns for the series shown in Fig 1A. Notes: The dark blue and dark red series represent the logarithmic return dynamics of the respective prices on the LME and SHFE. The vertical dashed lines denote the same as in Fig 1A.

Fig 1 illustrates the time variation of daily futures closing prices and corresponding logarithmic returns traded on the LME and SHFE, respectively, for four base metals over a period characterized by several significant economic, geopolitical, and health events. In Fig 1A, all prices have been converted to USD per ton (USD/ton). Based on the law of one price, similar price movements are observed for each metal between the LME and the SHFE. Nevertheless, prices in Shanghai are significantly higher than those on the LME, which may be attributed to trade barriers in the Chinese market [3].

Apart from zinc, which experienced significant price volatility, the price and return movements of the other three metals remained relatively stable prior to 2020. However, almost all metal prices plummeted after the onset of the COVID-19 pandemic in early 2020, followed by a sustained increase until the outbreak of the Russia-Ukraine war in 2022. Afterwards, prices further declined and fluctuated. The eruption of this geopolitical conflict introduced significant uncertainty, leading investors to worry about the security of mineral resource supply chains, which in turn triggered a rapid sell-off of assets. One consequence was that the price and return series of nickel became particularly distinctive, as evidenced by both the price series and the logarithmic return graphs.

Table 1 presents the summary statistics of the logarithmic daily return series. The mean values of all series are approximately zero. In terms of standard deviation, nickel exhibits the highest volatility, followed by zinc. Furthermore, the skewness and kurtosis suggest that most return series are left-skewed and leptokurtic in nature. Notably, LME nickel exhibits remarkably high skewness (5.21) and kurtosis (119.53), implying that extreme conditions likely occurred in the LME nickel market. Results from the Jarque-Bera (JB) test confirm that all return series significantly deviate from normality. Finally, we observe that all series are stationary, exhibit autocorrelation, and display ARCH effects. These results suggest that a TVP-VAR model with time-varying variances is suitable for modeling.

**Table 1:** Summary statistics of logarithmic daily returns.

	Cu_LME	Ni_LME	Al_LME	Zn_LME	Cu_SHF	Ni_SHF	Al_SHF	Zn_SHF
Mean	0.00032	0.00031	0.00023	0.00008	0.00028	0.00023	0.00024	0.00007
S.D.	0.0132	0.0257	0.0137	0.0162	0.0118	0.0215	0.0122	0.0141
Skew.	−0.3304	5.2189	−0.3420	−0.2236	−0.2798	−0.1802	−0.5552	−0.1279
Kurt.	6.0052	119.5347	8.7041	5.3837	6.5363	12.2958	6.6635	5.3412
J.B.	697.9*	1000000*	2433*	433.5*	944.8*	6379*	1080*	408.8*
ADF	−43.49*	−40.23*	−42.91*	−42.62*	−43.94*	−41.75*	−43.92*	−43.57*
PP	−43.49*	−40.21*	−42.92*	−42.62*	−43.90*	−41.87*	−43.90*	−43.60*
Q (20)	25.51	63.79*	20.42	33.48**	35.67**	36.60**	33.03	27.91
Q^2^(20)	112.40*	36.88**	304.94*	82.63*	269.20*	1156.00*	260.17*	155.73*

Notes: (1) SHF denotes the Shanghai Futures Exchange (SHFE). (2) Cu_LME, Ni_LME, Al_LME, Zn_LME, Cu_SHF, Ni_SHF, Al_SHF, and Zn_SHF represent log-returns of copper, nickel, aluminum, and zinc traded on LME and SHFE, respectively. (3) Mean and S.D. are calculated from logarithmic daily returns rather than price levels. (4) J.B.: Jarque-Bera test for normality; ADF/PP: Augmented Dickey-Fuller/Phillips-Perron unit root tests. (5) * and ** denote rejection of the null hypothesis at the 1% and 5% significance levels, respectively.

**Fig 2:**
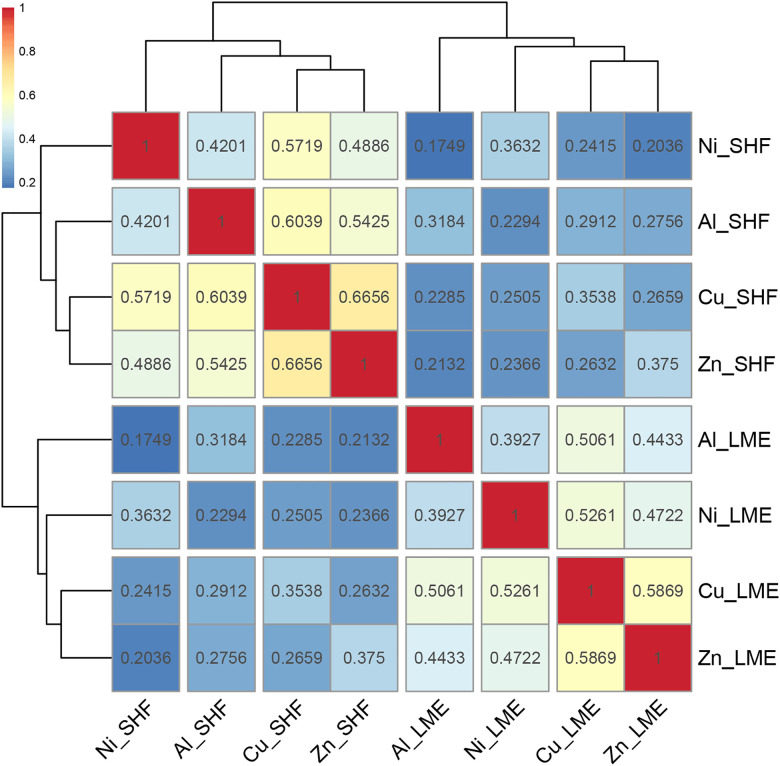
Hierarchical correlation clustering heatmap of LME and SHFE base metal returns. Notes: SHF denotes the Shanghai Futures Exchange (SHFE).

Fig 2 displays the hierarchical correlation clustering heatmap with Spearman rank correlation coefficients for all metal return pairs, reflecting the static monotonic relationships among them. The highest correlation is observed between SHFE copper and zinc, reaching 0.6656; next are the correlation between SHFE copper and aluminum, and between LME copper and zinc. Across both the LME and SHFE markets, copper demonstrates the strongest correlation with other metals. Conversely, the weakest correlation is observed between LME aluminum and SHFE nickel, with a coefficient of only 0.1749, followed by the correlation between LME zinc and SHFE nickel, and between LME nickel and SHFE aluminum. Nickel exhibits the weakest correlation with other metals in this analysis.

The distribution of warm and cool colors divides the graph into four relatively independent quadrants. More importantly, the cluster analysis results reveal that the LME and SHFE markets are grouped closely together, naturally forming two distinct clusters. These results suggest that, despite years of development and internationalization, the Shanghai base metals futures market remains relatively independent of the London market. This may be due to differences in investor composition, institutional frameworks, external economic and political environments, and varying levels of openness. However, it must be emphasized that this relative independence is observed only in a static and average sense.

## 4. Results and discussion

### 4.1. Averaged connectedness

We describe how specific markets transmit and receive spillover effects; in other words, how shocks to the LME influence the SHFE and vice versa. It is noteworthy that the average connectedness captures only the static spillover effects. Table 2 presents the results of the full-sample and frequency band analyses of directional, net, and total connectedness in the time-frequency domain. More specifically, Panels A, B, and C display the empirical results for the LME and SHFE in the full sample, short-term (1–5 days), and long-term (more than 5 days), respectively.

**Table 2:** Return spillover results in time and frequency domains.

	Cu_LME	Ni_LME	Al_LME	Zn_LME	Cu_SHF	Ni_SHF	Al_SHF	Zn_SHF	From
	Panel A: Time domain (in the full-sample period)								
Cu_LME	39.50	10.90	11.00	15.20	9.90	3.33	5.13	5.06	60.50
Ni_LME	12.40	49.00	6.71	9.93	4.80	10.50	3.19	3.53	51.01
Al_LME	13.50	7.84	50.70	10.50	4.34	1.88	7.50	3.80	49.31
Zn_LME	16.50	9.57	9.24	42.60	5.04	2.31	4.48	10.20	57.36
Cu_SHF	19.70	6.36	6.01	8.85	27.20	8.98	10.40	12.50	72.77
Ni_SHF	6.66	21.30	3.30	4.71	11.80	38.30	6.44	7.53	61.69
Al_SHF	7.87	3.74	13.50	5.69	13.70	6.77	37.40	11.40	62.63
Zn_SHF	8.59	4.85	4.90	18.10	14.30	6.98	9.88	32.30	67.66
To	85.10	64.50	54.60	73.00	63.90	40.70	47.10	54.00	TCI
Net	24.60	13.50	5.28	15.70	−8.87	−21.00	−15.60	−13.70	68.99
Net Subtotal	59.08				−59.17				
	Panel B: Short-term in the frequency domain (1–5 days)								
Cu_LME	32.40	8.73	8.71	12.30	8.15	2.66	4.09	4.12	48.71
Ni_LME	10.20	39.40	5.40	8.09	3.91	8.37	2.57	2.92	41.47
Al_LME	10.60	6.26	40.40	8.33	3.37	1.43	5.79	3.07	38.82
Zn_LME	13.30	7.94	7.08	34.60	4.01	1.87	3.38	8.23	45.78
Cu_SHF	14.60	4.65	4.35	6.51	21.30	7.06	8.01	9.65	54.85
Ni_SHF	5.06	15.00	2.51	3.56	9.37	29.90	5.07	5.97	46.51
Al_SHF	5.65	2.82	9.57	4.17	11.00	5.60	30.10	9.26	48.09
Zn_SHF	6.33	3.69	3.43	13.00	11.50	5.71	7.67	25.40	51.27
To	65.70	49.00	41.10	55.90	51.30	32.70	36.60	43.20	TCI
Net	17.00	7.57	2.23	10.10	−3.52	−13.80	−11.50	−8.05	53.64
Net Subtotal	36.90				−36.87				
	Panel C: Long-term in the frequency domain (more than 5 days)								
Cu_LME	7.08	2.15	2.27	2.95	1.76	0.67	1.05	0.95	11.79
Ni_LME	2.17	9.59	1.31	1.84	0.90	2.11	0.62	0.60	9.55
Al_LME	2.89	1.58	10.30	2.18	0.96	0.45	1.70	0.73	10.49
Zn_LME	3.21	1.63	2.16	7.99	1.04	0.44	1.10	2.00	11.58
Cu_SHF	5.08	1.71	1.66	2.34	5.89	1.92	2.40	2.80	17.93
Ni_SHF	1.59	6.35	0.79	1.15	2.39	8.37	1.37	1.56	15.19
Al_SHF	2.22	0.92	3.89	1.52	2.69	1.17	7.24	2.13	14.54
Zn_SHF	2.26	1.16	1.47	5.17	2.85	1.26	2.22	6.90	16.39
To	19.40	15.50	13.60	17.20	12.60	8.01	10.50	10.80	TCI
Net	7.63	5.96	3.05	5.57	−5.35	−7.17	−4.08	−5.61	15.35
Net Subtotal	22.21				−22.21				

Notes: SHF denotes Shanghai Futures Exchange (SHFE). The ‘From’ column represents the total connectedness that metal *i* receives from all other asset markets. The ‘To’ row denotes the total connectedness that metal *i* transmits to all other asset markets, while the ‘Net’ row indicates the net connectedness for each metal market. For example, LME copper’s total spillover effect on the SHFE market is 42.82% (19.70 + 6.66 + 7.87 + 8.59), with the short-term effect being 31.64% (14.60 + 5.06 + 5.65 + 6.33). The ‘TCI’ in the lower right represents the Total Connectedness Index, which measures the overall spillovers across the entire base metals market.

For the ‘To’ row, LME copper is the largest contributor of risk to the other markets, followed by LME zinc, while SHFE nickel and SHFE aluminum are among the smallest transmitters of risk to other markets. Furthermore, LME copper contributes 42.82% to the forecasting variance of the four non-ferrous metals (copper, nickel, aluminum, and zinc) on the SHFE, emphasizing its importance as a global pricing benchmark in the international market. Specifically, this implies that price volatility in LME copper can directly affect the price formation processes of SHFE metals, particularly in the short term (short-term effect accounts for 31.64%).

Analogously, according to the ‘From’ column, the largest receiver of spillovers is SHFE copper at 72.77% (of which 54.85% are short-term effects), followed by SHFE zinc at 67.66%. This aligns with the results in the ‘Net’ row: all metals in the LME market show a positive net spillover effect, with a Net Subtotal of 59.08% to the market (of which the short-term effect accounts for 36.90%). By contrast, the SHFE metals exhibit a negative Net Subtotal (−59.17% in the full sample and −36.87% in the short term), indicating that the SHFE acts as a net absorber of spillovers. Similar conclusions can be drawn from Panels B and C, with Panel B showing a larger proportion of short-term effects. These results clearly indicate that, overall, the London market functions as the price discoverer and net transmitter of shocks to the Shanghai market, while the Shanghai market serves as a net recipient. This pattern holds true across the full sample, as well as in both the short and long terms. The net spillover effect is more pronounced in the short term.

### 4.2. Dynamic total connectedness

In comparison to average connectedness, dynamic analysis contributes to a better understanding of the underlying relationships within the network, which is particularly beneficial for the in-depth exploration of the impact of specific events occurring during the sample period on spillover effects [27].

**Fig 3:**
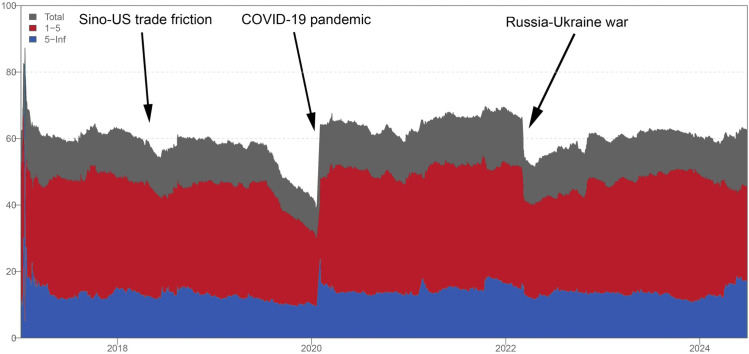
Dynamic total connectedness.

Fig 3 illustrates the dynamic total return spillovers of the LME and SHFE markets over time. Before 2020, the dynamic total connectedness remained relatively stable at approximately 60%. During this period, Sino-US trade friction emerged in 2018. In the era of global value chains (GVCs), Sino-US bilateral trade shocks could have multilateral effects, transmitting impacts to countries along these chains [28]. However, as shown in Fig 3, this significant economic conflict seems to have had a limited impact on spillovers between the London and Shanghai base metals futures markets. This may be attributed to the different pathways through which the Sino-US trade shock exerts its impact: initially affecting the global economy through supply chains, tariffs, and policy changes, but not directly transmitting to the metal futures markets. Although metals are one of the most important commodities in world trade, the nature of the base metal futures markets in London and Shanghai—such as price discovery and liquidity—may limit the extent to which their fluctuations affect each other, particularly in the face of external factors such as global economic growth, energy price volatility, technological advances, and the development of new materials. When the COVID-19 pandemic broke out in early 2020, the situation was entirely different, and the spillover levels between the LME and SHFE markets surged dramatically. This change stems mainly from concerns about disruptions in global resources and supply chains [20,29,30]. Following the outbreak of the Russia-Ukraine war in February 2022, spillover effects between the two markets initially plummeted and then gradually increased, which seems somewhat unusual. The possible reason for this phenomenon is that Russia and Ukraine are major global producers of mineral resources and energy, with Russia particularly dominant in metals such as iron, copper, nickel, aluminum, and zinc. The onset of the Russia-Ukraine war heightened anxiety about disruptions in the supply of critical minerals, leading to significant price volatility and risk spillover. After an initial strong shock, volatility spillovers gradually returned to normal.

From this, we can conclude that although the London and Shanghai markets are event-dependent, they exhibit distinct industrial characteristics. In other words, they are sensitive to major economic, political, and health events related to their own industries, while being relatively unresponsive to other occurrences. It is evident in Fig 3 that dynamic total connectedness is primarily driven by the short-term effects represented in the deep red areas, which is consistent with the results of Zhong et al. [31].

### 4.3. Time-varying net total directional spillovers

**Fig 4:**
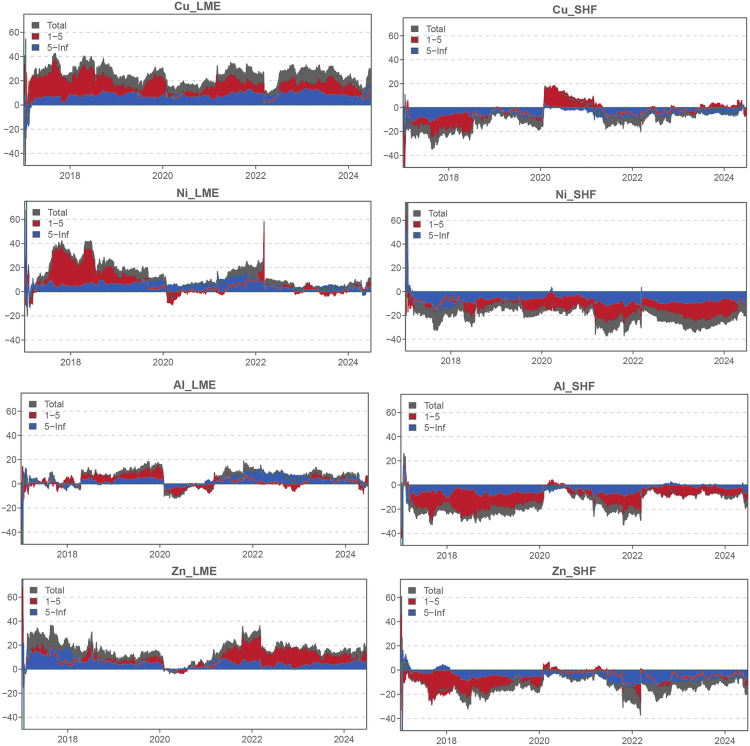
Time-varying net spillovers.

To identify the primary drivers of dynamic total connectedness, we determine the net transmitters and net recipients within the network (Fig 4). As shown in Fig 4, the London market has established itself as the dominant market for all metals throughout most of the sample period. This result contrasts with the findings of Kang and Yoon [2]. After examining the dynamic spillover effects between the Shanghai and London non-ferrous metal futures markets from 2007 to 2016, they concluded that the London market generally led the Shanghai market, particularly in the medium term. Nevertheless, for aluminum and zinc, the Shanghai market led the London market in the long run. In our study, however, the London market is a persistent net transmitter for both aluminum and zinc in the short and long terms, whereas the Shanghai market is a consistent net receiver. The decline in Shanghai’s market price influence observed after 2017 may be attributed to the combined effects of significant shifts in the global political and economic landscape following the Sino-US trade war in 2018, institutional frictions, along with a slowdown in China’s economic growth, a sharp correction in the real estate market, decreased infrastructure investment, and local governments’ debt resolution efforts. This divergence suggests that market leadership is contingent on both global events and domestic economic stability.

In terms of the price influence of individual metals, LME copper exerts the greatest impact, with a spillover effect of approximately 40% throughout the sample period, establishing it as the price leader in the base metals market. Following closely is LME zinc, which, except during the COVID-19 pandemic in 2020, exhibits spillover effects ranging from 20% to 30%. The metal with the most volatile spillover effects is LME nickel, which rose to 60% following the outbreak of the Russia-Ukraine war, far exceeding its usual level. This may be due to the fact that Russia is one of the major global nickel producers, and the war fueled concerns over supply disruptions. In contrast, aluminum and zinc also increased during the period, but to a lesser extent. This reflects the varying sensitivities of different metals to the same event, emphasizing the heterogeneity of base metals.

We also find that spillovers from LME nickel and zinc are primarily dominated by long-term effects after 2020. According to Chatziantoniou et al. [27], longer-term connectedness is more closely related to the structural changes during this period. For instance, the COVID-19 crisis in 2020 and the Russia-Ukraine war, which began in February 2022, may have altered the supply and consumption structures of nickel and zinc in international markets.

### 4.4. Time-varying net-pairwise directional spillovers

**Fig 5:**
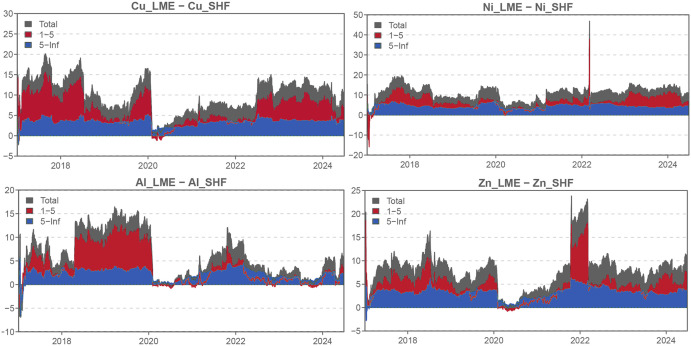
Time-varying net-pairwise directional spillovers.

To further analyze the price influence of the London and Shanghai futures markets, we develop a time-varying net pairwise directional connectedness graph (Fig 5). This graph illustrates the temporal evolution of return connectedness for the four base metals, offering a more granular view of dynamic return spillovers between the London and Shanghai markets. Remarkably, the four LME metals in Fig 5 continue to exhibit persistent net spillover effects on the SHFE market. That is, the SHFE market receives notable and consistent net spillovers from the LME market throughout the entire sample period. In particular, the net spillover effect of LME copper is not only large in magnitude but also long-lasting. It transmits substantial shocks to SHFE copper throughout the sample period, with the exception of a brief interval at the onset of the COVID-19 crisis. This further highlights the price leadership of LME copper. Turning to nickel, we observe that SHFE nickel received an abnormal short-term shock from LME nickel in early 2022, reaching approximately 47%. It is noteworthy that a significant event occurred in the global metal market in March 2022, known as the London Nickel Short Squeeze. This event was triggered by concerns over the supply of Russian Class 1 nickel and the global nickel supply crisis following the outbreak of the Russia-Ukraine war [32]. The incident led to a short-lived surge in LME nickel prices, reaching their highest level since 2008, followed by a rapid decline. This transmitted substantial volatility to the Shanghai market. It should be noted that the temporary market suspension (March 8–16, 2022) did not create a permanent discontinuity in our aligned daily return series, because non-trading days were excluded from the sample, and nickel trading resumed shortly thereafter. Furthermore, the 100-step forecast horizon used in the variance decomposition reduces the impact of this short-lived anomaly on the broader connectedness estimates, although the event remains clearly visible in the short-term nickel spillovers.

Regarding the dynamic net pairwise spillovers between LME and SHFE aluminum, significant net return spillovers (primarily short-term effects) were also observed from the London market to the Shanghai market between March 2018 and January 2020, a period during which Sino-US trade friction emerged. This supports the idea that the Sino-US trade conflict may have had a substantial impact on the aluminum market. Furthermore, the short-term spillover effects demonstrate that the market structure has remained unchanged, as previously discussed. For zinc, we observe that the LME zinc market exerts the strongest net return spillovers to the SHFE in early 2022, coinciding with the onset of the Russia-Ukraine war. The ongoing war has sharply intensified geopolitical tensions, leading to widespread uncertainty and heightened volatility in metals markets. As futures market investors must respond to rapidly changing circumstances and their potential impact on global stability, this environment amplifies the influence of market movements.

## 5. Concluding remarks

This study employs a TVP-VAR frequency connectedness framework to re-examine price spillovers between the LME and SHFE base metal futures markets from 2017 to 2024. The key findings and implications are summarized as follows:

First, the LME has reinforced its role as the global net transmitter of price spillovers, while the SHFE remains a net receiver. This dynamic is particularly pronounced in the short term, underscoring the rapid transmission of shocks from London to Shanghai. The observed decline in the SHFE’s price leadership relative to its historical position challenges the narrative of uninterrupted financial internationalization. This shift appears driven by the confluence of global geopolitical turbulence and domestic Chinese challenges, including an economic slowdown and structural adjustments in the real estate sector, reduced infrastructure investment, and local governments’ debt resolution efforts.

Second, market sensitivities are highly event-dependent and metal-specific. The LME’s influence surged during supply chain crises (e.g., the 2022 nickel short squeeze), whereas the SHFE’s responsiveness appears to be moderated by institutional barriers. In this context, these institutional barriers primarily refer to frictions that limit the SHFE’s integration with global benchmark pricing, including differences in trading rules and contract specifications, restrictions on cross-border participation and arbitrage, differences in investor composition and trading hours, and relatively lower transparency of inventory and related market information. Furthermore, the spillovers of nickel and zinc are dominated by long-term effects post-2020, suggesting that the COVID-19 pandemic and the Russia-Ukraine war have induced lasting structural changes in their supply and demand fundamentals. In contrast, the Sino-US trade conflict had a more discernible impact on the aluminum market.

These findings carry salient implications for policymakers and market participants. For emerging economies, enhancing market resilience and global standing necessitates coordinated efforts in three key areas: regulatory coordination to reduce institutional frictions and better align trading rules with global benchmarks; market development to strengthen price discovery mechanisms and deepen the domestic investor base; and enhanced transparency through the more timely disclosure of market-related and inventory information. Furthermore, the high sensitivity of metals like nickel to geopolitical crises underscores the strategic importance of establishing physical commodity reserves to mitigate supply shocks.

This study lays a foundation for further exploration, although it acknowledges certain limitations. The research primarily focuses on the four most actively traded base metals and the bilateral linkage between LME and SHFE, which could be expanded in future investigations. Subsequent research should consider incorporating a broader array of commodities—including energy and precious metals—and examine multi-market network interactions. Additionally, integrating Environmental, Social, and Governance (ESG) metrics into connectedness models presents a promising strategy for understanding how the global transition toward green energy is reshaping interdependencies within the metal markets.

## Supporting information

S1 Data Data and R code (for PLOS ONE). (RAR)
